# Supplementary material for: A Systematic Review of Treatment Outcome Predictors in Deep Brain Stimulation for Refractory Obsessive-Compulsive Disorder
Source: Brain Sci. 2022 Jul 17;12(7):936. doi: 10.3390/brainsci12070936 (PMC9316868; doi:10.3390/brainsci12070936)
Supplement: Supplementary file 1 [file brainsci-12-00936-s001.zip › brainsci-1763284-supplementary.pdf]

Table S1. Searching strategy of each database.

Databases	Search Keywords
Scopus	TITLE-ABS-KEY ("obsess*" OR "compuls*" OR "OCD") AND TITLE-ABS-KEY ("deep brain stimulation" OR "DBS") AND LANGUAGE (english)
PubMed	(obsess*[Title/Abstract] OR compuls*[Title/Abstract] OR OCD[Title/Abstract]) AND (deep brain stimulation[Title/Abstract] OR DBS[Title/Abstract]) Filters: English
Embase	#3 #1 AND #2 AND [english]/lim<br>#2 'deep brain stimulation':ti,ab,kw OR dbs:ti,ab,kw<br>#1 obsess*:ti,ab,kw OR compuls*:ti,ab,kw OR ocd:ti,ab,kw

Table S2. NHLBI Quality Assessment Tool for Before-After (Pre-Post) Studies With No Control Group.

#	Raymaekers, S., et al.	Mallet, L., et al.	Graat, I., et al.	de Koning, P.P., et al.	Haq, I.U., et al.	Schüller, T., et al.	Welter, M.L., et al.	Hartmann, C.J., et al.	Balderman, J.C., et al.	Li, N., et al.	van der Vlis, T., , et al.	Mosley P.E., et al.	Chen, X., et al.	Widge, A.S., et al.	Liebrand, L.C., et al.	Germann, J., et al.	Sildatke, E., et al.
1. Was the study question or objective clearly stated?	Yes	No	Yes	Yes	Yes	Yes	Yes	Yes	Yes	Yes	Yes	Yes	Yes	Yes	Yes	Yes	Yes
2. Were eligibility/selection criteria for the study population prespecified and clearly described?	Yes	Yes	Yes	Yes	Yes	Yes	Yes	Yes	Yes	Yes	Yes	Yes	Yes	Yes	Yes	Yes	Yes
3. Were the participants in the study representative of those who would be eligible for the test/service/intervention in the general or clinical population of interest?	Yes	Yes	Yes	Yes	Yes	Yes	Yes	Yes	Yes	Yes	Yes	Yes	Yes	Yes	Yes	Yes	Yes
4. Were all eligible participants that met the prespecified entry criteria enrolled?	NA	NA	NA	NA	NA	NA	NA	NA	NA	NA	NA	NA	NA	NA	NA	NA	NA
5. Was the sample size sufficiently large to provide confidence in the findings?	Yes	No	Yes	No	No	No	No	No	Yes	No	No	No	No	No	No	No	No
6. Was the test/service/intervention clearly described and delivered consistently across the study population?	Yes	Yes	No	Yes	Yes	Yes	Yes	Yes	Yes	Yes	No	No	Yes	Yes	Yes	Yes	Yes
7. Were the outcome measures prespecified, clearly defined, valid, reliable, and assessed consistently across all study participants?	Yes	Yes	Yes	Yes	Yes	Yes	Yes	Yes	Yes	Yes	Yes	Yes	Yes	Yes	Yes	Yes	Yes
8. Were the people assessing the outcomes	NA	NA	NA	NA	NA	NA	NA	NA	NA	NA	NA	NA	NA	NA	NA	NA	NA

blinded to the participants' exposures/interventions?																	
9. Was the loss to follow-up after baseline 20% or less? Were those lost to follow-up accounted for in the analysis?	No	No	No	Yes	Yes	Yes	Yes	Yes	Yes	Yes	No	Yes	Yes	Yes	Yes	Yes	Yes
10. Did the statistical methods examine changes in outcome measures from before to after the intervention? Were statistical tests carried out that provided p values for the pre-to-post changes?	Yes	Yes	Yes	Yes	Yes	Yes	Yes	Yes	Yes	Yes	Yes	Yes	Yes	Yes	Yes	Yes	Yes
11. Were outcome measures of interest taken multiple times before the intervention and multiple times after the intervention (i.e., did they use an interrupted time-series design)?	Yes	Yes	Yes	No	Yes	Yes	No	No	No	No	No	Yes	No	No	No	No	Yes
12. If the intervention was conducted at a group level (e.g., a whole hospital, a community, etc.) did the statistical analysis take into account the use of individual-level data to determine effects at the group level?	NA	NA	NA	NA	NA	NA	NA	NA	NA	NA	NA	NA	NA	NA	NA	NA	NA
Rating	Good	Fair	Fair	Fair	Good	Good	Fair	Fair	Good	Fair	Poor	Fair	Fair	Fair	Fair	Fair	Good
